# Extracts and Gleanings

**Published:** 1873-05

**Authors:** 


### PART II.

### EXTRACTS AND GLEANINGS FROM OUR EXCHANGES.

### *MULTUM IN PARVO.*

CLINICAL SIGNIFICANCE OF SPUTA.—Dr. A. J. Steele, of St. Louis, writes to the *Medical and Surgical Journal* of that city:

We have not been able to diagnose pulmonary tuberculosis by means of the tubercle cell found in sputa; and while willing to believe that there is a tubercle cell, *sui generis*, which, if found in expectorated matter, would afford sufficient evidence of the presence of tubercle in the lung, still, practically, this is not known to be possible; for there are so many other elements going to make up the mass of the sputa, namely: epithelial, pus and mucous cells, granular matter, broken-down and degenerated tissue, that our cell tubercle, if actually present, is so obscured and concealed as not to be recognized; again, if present, it has so undergone disintegration as to have lost its characteristics, and thus cannot be made available.

The microscope detects in expectorated matter but a single substance that may be considered pathognomonic, namely: elastic tissue. Its presence signifies lung destruction, brought about by inflammation and ulceration, the latter process not confining itself superficially to the mucous surface, but having extended deeper into the parenchyma itself.

The basement framework of those organs so beautifully adapted to the aeration of the blood, reminding one, with their open spaces and interstices, of sponge, is composed of elastic tissue, which, being of comparatively low vitality, *i. e.*, less vascular than most other tissues, is but slowly susceptible to the destructive process; and thus, from ulcerative action, fragments of it will be detached and thrown off, still retaining, however, their histological arrangement, being thereby readily recognized under the glass. All sputa does not contain it, but, when found, it points unerringly to but one source, and thus its clinical importance.

While the appearance of these elastic fibrillæ is peculiar, yet the novice might confound them with the fibre of linen, silk or cotton. Still, each have their characteristics, and a little practical observation will enable one to distinguish each from the other. So, also, the curved streaks of mucus so often present have been mistaken for elastic fibres. A drop of acetic acid at once desides the differerence. Elastic fibres are curved, divide dichotomously, are of equal width, sharply contoured, and remain unaffected by acetic acid; once seen, are ever after recognized; best studied from section of healthy lung.

If from the sputa is taken a small quantity of the paler portion of the lumps, and if the grayish masses are especially examined on the slide, the elastic tissue will ordinarily be found. Specimens from different portions of the sputa should be subjected to the glass; a delicate hook to catch up, and scissors curbed on the flat to nip off the matter examined, are convenient. A drop of acetic acid, which dissolves for the most part the substances present, brings out the elastic fibres more decidedly. When much in doubt, it is well to boil the suspected expectoration in *liquor sodæ.* On cooling, the elastic tissue falls to the bottom of the settling glass, and can readily be removed with the pipette. A power of three hundred is quite sufficient to exhibit the fibres.

Such a simple matter is this examination, that I am in the habit of subjecting the expectorated matter of most patients afflicted with lung trouble to it.

The special practical importance of this sign is its early recognition of the destructive changes taking place, before other signs indicate it; before even it is suspected, the microscope tells the certain story of disintegration of air vesicle walls. If diagnosis is much in the practice of medicine, surely an early knowledge of the facts is all the more important. "Had I seen the case earlier, or had I only known in time," so often remarks the medical man, "the issue would have been different." While undue prominence should not be given to the microscope, still it should hold its legitimate place in the means employed to diagnose disease. Too much neglected in the past, the day comes when it will be found a working tool in the hands of every physician.

Lung destruction associated with tuberculosis is much less amenable to treatment than when it is simply an inflammatory affection. The concomitant symptoms in the former case, the hereditary and personal peculiarities, with the history, enables it to be readily distinguished, and makes the prognosis guarded; while in the latter case a good result would be expected, even though a cavity in the lung should have been found. Just here, and in conclusion, we cannot avoid the suggestion that old cicatrices found in post mortem examination of the lungs, and proclaimed to be evidence of pulmonary consumption cured or arrested, have been proof merely of nature's repair of excavations produced by a simply ulcerative process entirely unconnected with the tubercular diathesis. These become the cases of so-called "spurious consumption."—*Chicago Medical Examiner.*

The Treatment of Prolapsus Uteri Without Mechanical Means.—Nicolai Andreef, of Kasan, states that he remarked in some cases of disease of the uterus that the application of tincture of iodine restored the relaxed and weakened ligaments to a nearly normal state. In consequence of this observation, he determined to try its action in cases of complete descent and prolapse of the uterus. The first trials gave such favorable results that he was led to investigate its action systematically, and his results have proved so satisfactory that he now publishes them in the hope of inducing others to give the results of their experience with it. The following is one of his cases: In August, 1871, a patient who had suffered for four years from complete prolapsus uteri came under his care. She had tried various mechanical means for obtaining relief, without advantage. He prevailed upon her to submit herself to his treatment, and in four weeks he dismissed her cured. She was twenty-two years of age, emaciated and of weak constitution. The prolapse had followed the birth of a child four years previously. The descent of the uterus was so easy that when replaced a strong cough brought it down to nearly its whole extent. The treatment pursued consisted in replacing the uterus whilst the woman was in a recumbent position. Then, with the aid of the speculum, the fold of the vagina—that is to say, the part surrounding the os—was painted with half a drachm of a tincture composed of one part of tincture of iodine and one part of alcohol. He diluted the officinal tincture of iodine because the undiluted tincture sometimes sets up acute catarrh of the vagina and even of the uterus; as he had had an opportunity of observing previously. After the application of the tincture, the patient remained for three days in bed, and had an injection four times a day of pure spring water, at a temperature of 77° F. The painting was then reapplied and the douches repeated. After a repetition of this plan of treatment four times, the patient found herself well, and was dismissed. Four months subsequently, she was pregnant and quite healthy; no descent of the uterus had occurred. He gives the details of other cases in which the prolapse was both complete and incomplete. His experience has taught him that the best results are obtained when the following conditions exist: 1. The uterus must be capable of being replaced in position. 2. Before the iodine plan is adopted, all other diseases and lesions of the vagina or uterus, such as erosions, ulcerations, &c., must as far as possible be removed, otherwise inflammatory reaction is apt to take place. 3. It is only requisite to paint the vault of the vagina, and at the commencement of the treatment with dilute solutions and weak doses. Subsequently, however, stronger solutions may be employed. Cold vaginal douches must always be used after the application of the iodine, with a view of preventing inflammation of the uterus and vagina. 4. In the majority of instances, it is not requisite for the patient to remain in bed after the first two applications. After ten days she may be allowed to return to her work, if not of too severe a character. 5. It is important that the bowels should be thoroughly cleared. 6. The interval between two successive paintings should not be less than three days; also, with the object of preventing irritation of the vagina and uterus, the cold douches may be continued for some time after the last application of the iodine. When the cure is complete, the vagina does not become narrower, but it is thicker than it was before. After reposition of the uterus, some sympathetic disorders of the stomach often occur, but this is easily remedied.—*The Clinic, from Virchow's Archiv., Band lv*, 1872.

THE EXTRACTION OF FOREIGN BODIES FROM THE EXTERNAL EAR.—Dr. Jos. Gruber (*All. Wien. Med. Zeit.*, 15th October) says that since empiricism was abandoned in treatment of diseases of the ear, physicians have become convinced that the extraction of foreign bodies from the external ear should almost always be made by injections, and that it is only in the very rarest cases that instruments should be made use of. The external ear is so constructed that in the way in which ordinarily foreign bodies are introduced into it—which, as is well known, takes place in children during play—the body introduced does not go far in, and is so loose that it can be brought away by an ordinary injection. Where the cartilage joins the bony part of the ear, the diameter of the canal is narrowest, whereby the entrance of larger bodies into the deeper portion is much hindered; besides which, the axis of the auditory passage is bent at an acute angle, and the corner of the angle takes place at the narrowest point. In this peculiarity we must seek the cause why large foreign bodies get beyond the cartilaginous portion into the deepest section of the passage, and, therefore, in the majority of cases, can be very easily extracted. Notwithstanding all this, it is by no means rare to find that dangerous results take place from attempts to remove such bodies by all sorts of instruments. Hair-pins, pincettes, and even scissors are often used in the most dangerous way to attempt extraction, which may, any of them, cause dangerous accidents to the patient. Such operations have the further disadvantage, that they make extraction by simple injections more difficult; so that an ear doctor is always glad to hear that the patient has not been meddled with until brought to him for inspection.

The washing out of the auditory passage, if it is to be made well and the end well considered, is by no means so simple and easy as it appears in general; and persons who are well acquainted with the art will confess that this requires as much practice as catheterism of the Eustachian tube, and that in order to arrive at the goal by injection, he that is best acquainted with the anatomy of the parts is most likely to succeed.

It is best in all cases, unless the foreign body be likely to do injury through its chemical properties, to let it alone until any inflammation caused in instrumental interference be a little calmed, before we commence injection to remove it. A boy at school put a small foreign body into his comrade's ear, and pushed it deeply in by means of his pencil. Pain soon after came on, and a physician, after attempting extraction by means of instruments, fruitlessly, prescribed an anodyne medicine. Five weeks after, the patient visited Dr. Gruber, much brought down in health, and feverish. The parts about the right auditory passage were much swollen, inflamed, and the passage thereby much narrowed and filled with exudation. After removing the latter, there was seen, deep down, a dark brown body, which lay very deep. The washing out of the passage was likely, in this case, to be useless, and we confined ourselves to cause the inflammatory processes to disappear first of all; and then, when the auditory meatus was larger, to use injection. To do this, a wash of morphia and acetate of lead was used, and cold compresses were ordered behind the ear, and the cleansing of the auditory passage by injections with tepid water was used once a day. During the injections a portion of the water from the very first came through the nostrils, which showed that the membrana tympani was at any rate perforated.

After the symptoms of inflammation had disappeared, the foreign body was also more clearly seen, but its extraction by washing out of the auditory passage was not yet possible, so that small pieces of compressed sponge were inserted in order to widen the passage; and a solution of sulphate of zinc (five grains to the ounce) was dropped into the ear. In the month of December—that is, after a stay of the foreign body for three months—it came away after a simple injection of tepid water. The issue of pus had ceased for some weeks, although the foreign body was still *in situ*, and when that was removed, the membrana tympani was seen degenerated like a cicatrix, but not perforated. The perforation had healed up whilst the foreign body was still in the ear.

Diagnosis of Tuberculosis (Dr. M. Heitler, Vienna: *Wiener Mediz. Presse*, October 6, 1872).—The diagnosis of tubercle is not ordinarily difficult; the appearance of the patient and the history of the case often render an examination superfluous, excepting for the purpose of a more thorough knowledge of the extent of the ravages of the disease in the lung. Every physician of experience, however, knows that there are some cases in which it is difficult to say whether tubercle is present or not in the diseased lung. The disease does not always occur in its characteristic form, but in a group of symptoms by which we may often be misled. Not only miliary tubercle offers no sure means of recognition, but also the subacute and chronic forms present many difficulties to the diagnostician, in their stage of development, by a group of symptoms which belong also to other diseases, and which mask the tubercular nature of the disease in question.

Very frequently a patient comes complaining of palpitation of the heart, a weak stomach, and other digestive troubles. He says "he has no disease of the chest, and has never been troubled by a cough," etc.; but if we continue our questions, we discover that in many cases a slight weakness has been noticed by the patient, and that he has commenced to grow thin, he knows not why, and that he has night-sweats; others say they have noted none of these latter symptoms, but that they suffer from palpitation of the heart, dyspepsia, etc., and they think they have heart-disease or disease of the stomach.

If we examine such persons, we find nothing abnormal in the organs of circulation or digestion, but we may find a marked dullness in the apices of the lungs. However, not only symptoms characteristic of other diseases may render the diagnosis of tubercle difficult, but the pulmonary symptoms themselves may at times render the diagnosis of tubercle doubtful or improbable. It sometimes happens that the tubercular disease is ushered in under the form of a broncho-blennorrhœa with copious mucous expectoration, and in the lungs we find symptoms of catarrh only.

In such cases, however, the marked increase in the emaciation, the weakness and continued fever, lead us naturally to a repeated examination of the chest, and very soon we discover dullness on percussion, with other characteristic symptoms.

The most difficult cases are those where we have to diagnose between a croupous pneumonia undergoing resolution, and a tuberculosis. It is often impossible at the first examination to decide the nature of the disease; and a period of observation extending over several days is necessary in order to make a positive diagnosis.

In other cases we can at once, by means of the physical signs and the history of the case, establish the diagnosis. If, for example, we find a symmetrical dullness extending over the entire lung, accompanied by a consonating subcrepitant râle, or bronchial breathing, with fever, and if we discover that the disease has reached that point, or passed it, when, if it were an ordinary pneumonia, resolution should have commenced—*i. e.*, the beginning, or, at latest, the middle, of the second week—we are justified in supposing that we have to deal with a case of tuberculosis, and not with one of pneumonia in the stage of resolution.

I lay special stress on the similarity of the physical signs found throughout the tubercular lung; whereas in the lung of a patient affected with pneumonia we may find fine vesicular breathing at the base, while above there are various râles and bronchial respiration. In tuberculosis, the part first attacked does not become free as the process of disease spreads throughout the lung, and at last we may hear the same sounds at all points in the lung.

There are other cases in which a positive diagnosis can only be made from the history and the course of the disease. Suppose we find in the upper portions of the lungs a dullness, with consonaiing subcrepitant râles, confined either to a very small spot, or extending over the greater part of the superior lobe of the lung. If the individual is anæmic, and states that he has already had fever and a cough for several weeks, and that the latter preceded the fever, and that emaciation has been slowly progressing, we may safely conclude that the physical signs indicate the commencement of a tubercular infiltration, and not the resolution of a croupous pneumonia.

In many cases the history and the appearance of the patient furnish no sure guide to a diagnosis of tubercle. In many cases the individual has been perfectly well, has a well-formed thorax, and looks pretty well at the time the diagnosis is made. In such a case only a consideration of the physical signs attending a pneumonia in an advanced stage of resolution, and the course of the disease, can render our diagnosis beyond doubt. In a few days the diagnosis will become clearer; the circumscribed dullness, which sometimes remains stationary quite a long time, will suddenly spread; the fever will continue very high, and the patient will rapidly fall into a state of collapse, with all the symptoms of well-defined tuberculosis. Dr. Heitler will continue his reflections upon this subject, which he has ample opportunity for studying in the wards of the "Allgemeines Krankenhaus" in Vienna.—*Medical Times.*

Hooping-Cough.—Purgation with calomel; if febrile symptoms, calomel and antimony; an occasional emetic, and small and repeated doses of carbonate of potassa, or the following formula: Potassæ carb., ʒj.; coccus cacti, gr. x.; aq. fervent, q. s. The dose according to age; for an infant, a teaspoonful thrice daily.

RHUS TOXICODENDRON.—Dr. Scudder (*Eclectic Medical Journal*) writes: I have employed the rhus in quite a number of cases during the past five years, and find it a very reliable remedy—a true specific, if the diagnosis is well made. The characteristic symptom indicating its use to me is a peculiar glistening redness of skin, with unpleasant burning. In fever and pneumonia it sometimes shows itself on one cheek. In remittent fever, and diseases of the chylopoietic viscera, it may show above the frontal and orbital sinuses. Occasionally it is seen about the mouth, attended with swelling of the lips, and very unpleasant "fever" blisters. I have noticed it first on the nates in protracted diseases, and in some cases on the extremities.

One of my first cases, some five or six years ago, presented the usual symptoms of continued fever, but with marked irritability of the nervous system. The sedatives were employed, with gelseminum, the use of the bath, and associate means, for ten days, without any effect. The patient complaining of pain and burning in the foot, I examined it, and found about *half* the foot swollen and of this glistening red color. I called it erysipelas, but it was not. Whatever it was, the disease yielded readily to rhus, gtts. v.; water, ℥iv.; a teaspoonful every two hours, alternated with Aconite. In forty-eight hours the patient was convalescent.

I may name as an additional symptom the same bright, glistening redness of the tongue, with anterior papilla presenting the peculiar appearance of the little bubbles upon the surface.

Was called to see Miss G., æt. seven, in October. She had been suffering with swollen face and burning pain for the week past. There were a few vesicles about the mouth, and the characteristic shining appearance above named. Was not much sick at first, but now complained of pain in back and limbs, and had some fever.

Prescribed—℞. Tinct. rhus, gtts. iv.; water, ℥iv.; a teaspoonful every three hours—no other medicine. Was entirely relieved by the second day.

Mrs. L. has had a miscarriage, and the fifth day was attacked with facial erysipelas. The fever runs high, with irritability of the nervous system and sleeplessness. The face shows three points of disease, which are spreading rapidly. They are swollen, bright red, and very glistening. She complains of burning and pain.

Prescribed—℞. Tinct. rhus, gtts. v.; tinct. aconite, gtts. x.; water, ℥iv.; a teaspoonful every hour. Paint the parts every four hours with tinct. veratrum, ʒj.; dilute alcohol, ℥j.; keeping them covered meanwhile with lard.

Though the disease was a severe one, it yielded to this treatment, and the erysipelas stopped on the third day, the patient making a quick recovery.

In two cases of this epidemic throat disease, I have noticed this redness and swelling of the face. One would have been pronounced erysipelas. In both, rhus gave prompt relief.

McB., æt. thirty-two, was attacked with severe sore throat early in November; has had occasional attacks of quinsy. Found the fauces, tonsils and pharynx vividly red, tumid, dry, with the peculiar shining appearance named. Prescribed rhus, alternated with aconite. Was out looking after his business the third day afterwards.

Belladonna in Ophthalmia.—Mr. Henry Power writes to the *Practitioner:*

The following is a *résumé* of one of the cases that has recently been under my care, showing the benefit that may be derived from the use of the extract of belladonna in obstinate phylctenular ophthalmia:

M. C., æt. four, was brought to me in the beginning of January of the present year. She had had measles (not an unusually severe attack) in August, 1871; this was followed by stoppage in the nostrils and herpetic eruption on the lip and on the right ear. At the end of August her mother took her to Yarmouth for a month. She was ailing all the time she staid there, complaining of thirst, with restlessness at night, languor, and loss of appetite. These symptoms continued up to November, when the right eye became affected. She was taken to an ophthalmic hospital, when some tonic medicine was ordered for her, and a poultice was directed to be applied. A crop of pustules appeared over the brow, temple and cheek, and the left eye became affected. I then saw her, and found that she was suffering from a well-marked attack of phylctenular ophthalmia. On examining the eyes, several pustules were visible on the conjunctiva at the margin of each cornea, whilst superficial abrasions or ulcers were apparent on the corneæ themselves, the right being much the more severely affected, so that a large part of its surface was cloudy. She was ordered a purge, and a powder containing three grains of hydrargyrum c. cretâ with one of quinine, thrice daily. A four-grain solution of atropine was dropped in, and a lotion of extract of belladonna given to take home, and to be used frequently. But little improvement resulted, though the mercury and quinine were continued for a fortnight. The bowels being somewhat disordered, and the lips and ala nasi having some pustules upon them, it was thought likely that there were worms. Another purge of compound jalap powder and a turpentine injection were therefore ordered. No worms, however, were observed in the motions; cod-liver oil was now prescribed, and after a short time a little steel wine was added to it. The use of belladonna lotion was continued. This plan was persevered in for a month, with little benefit, the child sometimes opening the eyes in the evening and playing about, but always coming to me in the morning with intense photophobia. The mother was exceedingly intelligent, and assured me that the bowels were regular, and that the food given to the child was carefully selected, wholesome in quality, and though its appetite was variable, yet, upon the whole, the quantity was sufficient for its needs. Calomel powder was now applied directly to the cornea for several days, but ineffectually, so far as the relief of the photophobia was concerned.

Finding that the symptoms were stationary, I now determined to tap both eyes with a broad needle, and allow the aqueous humor to escape. The benefit was considerable, the child opening the eyes on the following days for some hours, and I thought recovery would take place without further trouble. However, a relapse soon took place, and she was now placed upon one-sixth of a grain of good extract of belladonna three times a day. In two days the mother brought her to me obviously greatly improved. The intolerance of light had greatly diminished, and she even saw to pick up a pin on the carpet. She continued the use of the extract for a fortnight, when it was given up and some quinine mixture ordered. A few days later she went into the country. This child has lately been seen by me, looking fresh and rosy, with only slight nebulæ on the corneæ of both eyes, but able to bear strong light perfectly; and I can feel no hesitation in attributing the improvement here observed to the internal use of the belladonna, it was so immediate and proved so persistent.

Ergotine as a Hemostatic.—Dr. C. H. Boardman, (*Northwestern Medical and Surgical Journal*), reporting a case of placenta previa, remarks:

When I first saw my patient at this time, she was lying down, pale and quite faint; a hemorrhage of about a half pint having ceased just before. An examination established the fact that a placenta previa was to be dealt with, and stringent orders were enjoined, such as were appropriate to that condition. The administration of *Ol. Erigeron Philadelphicum* proved efficient for several days in checking the flow as soon as it showed a disposition to recur; and opium occasionally as a suppository both relieved the pelvic pains, now commencing, and also seemed to aid in suppressing the discharge. The beginning of the ninth month was reached thus in safety, and the bleeding seemed so fully under control that I began to entertain hopes of my patient's accomplishing the full time. But she had found her enforced quietude very irksome, and believing that the hemorrhage was now completely arrested, she yielded to her urgent desire to enter again upon some, at least, of her household duties.

Flowing commenced at once, and to a serious extent; former remedies were no longer useful, and I resorted to ergotine. Having no means for measuring it at hand, I poured about two drops into water enough to render it sufficiently fluid for my purpose. Half of this was injected over the biceps, and in a few minutes, not more than five, the patient spoke of a giddy, throbbing sensation in her head, and the flow of blood ceased abruptly, and as completely as though a tampon had been used. From fifteen to eighteen hours later its threatened return was again as promptly checked, and for some ten days it was completely held in abeyance by this remedy occasionally administered, aided, of course, by absolute quiescence, suitable diet and other means appropriate as adjuvants. Thus, for a period of nearly two weeks, the perils incident to this very grave condition were averted, and the patient brought safely to within a fortnight of her full time, where all other resources had failed, and where, but for the ergotine, she must have encountered greater dangers by so much as the enforced confinement might have anticipated the natural period for that event.

It may be said, in conclusion, that a week or ten days before uncomplicated labor would have occurred, hemorrhage set in, so violent as to defy all efforts for its suppression; the os dilating, had torn away from quite a large segment of the placenta on the left side. The head presenting in the first position, after separating the placenta to a sufficient extent, the forceps were applied, my friend Dr. W. assisting, and the child quickly delivered. It was a male, very large and well developed, but completely asphyxiated by a double coil of the cord about its neck, so as to resist long and vigorous efforts for its resuscitation.

The mother, though reduced and exhausted by loss of blood, especially during labor, to such an extent that fears were entertained as to her recovery, reacted shortly, and, possessing remarkable recuperative powers, was again engaged in her household duties in two weeks, and showed no trace of the dangers through which she had so recently passed.

SPINA-BIFIDA.—Dr. Wilson, of Glasgow, relates a case of spina-bifida which he treated thus: The tumor being moistened over with carbolized oil, was opened by a free longitudinal incision, under an antiseptic veil of surgeon's lint soaked in carbolized oil. The lint was removed, and a large piece of carbolized lac plaster applied, and over this another, kept in position by adhesive plaster, one edge being left comparatively free for escape of fluid. A soft folded handkerchief was laid over all. A good firm pad of skin resulted.

Ergot in the Treatment of Cystitis with Hematuria. Dr. W. F. Alexander (*Medical and Surgical Reporter*) says:

In compliance with your request that country practitioners shall give you the benefit of their experience and opinions, and with a desire to have thoroughly tested the therapeutics of a remedy that I am satisfied is of inestimable value, I report the following case:

I was hastily summoned, after dark, October 18th, 1872, to see Mr. J. S., a gentleman aged seventy-two, who had fallen on his porch, unable to get into his house without assistance.

I found him suffering excruciating pain, referred to perinæum and pubic region, voiding from the bladder clots of blood. Ordered a hot bath, after which I introduced the catheter, bringing away about half a pint of very bloody urine; administered (internally) chloroform and left him sleeping quietly and free of pain.

Called the next morning, October 19th. Full bounding pulse; skin hot and dry; voiding small quantities of very bloody urine incessantly, accompanied with great pain and with tenderness upon pressure over the pubic region. There was nothing to lead me to believe that the kidneys were implicated; the trouble was with the bladder, and with that alone. It was a pure case of idiopathic cystitis with hematuria. I gave—℞. Ant. et. pot. tart, gr. $\frac{1}{6}$; sulph. morphiæ, gr. $\frac{1}{4}$; elm water, ℥j. To be repeated every three hours unless symptoms abated and my patient slept, in which event he was not to be roused. Drink slippery elm water; diet strictly antiphlogistic.

This treatment was continued several days, and my patient improved, the urine becoming free of blood and passing without pain, appetite, strength, etc., good. The bowels, however, had not been moved for several days, and I directed an enema of cold water and salt. Instead of this, my patient took a tablespoonful of ol. ricini, and this not bringing about the desired result, he repeated the dose some three or four times, which brought on violent purging, straining, and all his former symptoms greatly aggravated.

I now gave everything calculated, in my opinion, to meet the emergency. I will not, for fear of proving prolix, enter into detail, but give the treatment in general terms. Ant. et. pot. tart., Norwood's tr. verat. virid., bi. carb. pot., etc. Injections into the bladder of sol. per. sulph. ferri, also acetic acid. Applied ice over the pubic region and perinæum, also introduced continually (or rather made the patient do so) small portions up the rectum.

All my efforts proved futile; my patient grew worse; true, I was enabled to relieve his suffering by the administration of morphia, chloroform, etc., but the disease was triumphant, the quantity of blood was not in the least diminished, and my patient was rapidly succumbing to the drain upon his system, and showing signs of anæmia. I now gave—℞. Fluid ext. ergot, gtt. xx.; tr. ferri chlo., gtt. x.; to be repeated every three hours.

In twenty-four hours the quantity of blood in the urine was greatly reduced, and in thirty-six had entirely disappeared; on the third day after the administration of the ergot, my patient was convalescent. I saw him to-day, a week since I discontinued my visits, and there has been no return of the disease; the ergot, however, had been continued three times a day.

In this case I had the valuable assistance and advice of Drs. Tanner and Reynolds, gentlemen of ability and experience, and they express themselves highly pleased with the action of the ergot.

I should not neglect to add that my attention was attracted to ergot as a general styptic by your valuable journal.

I am thoroughly satisfied, in all cases requiring an astringent to blood-vessels, it will prove a true friend that will not desert you in the hour of need.

SICK HEADACHE (*British Medical Journal*, December 21, 1872). In a report on the treatment of this distressing affection, Dr. Wilks recommends three remedies. To cut short an existing attack, he gives bromide of potassium in doses of gr. xv. to xx. One dose is often sufficient. As a a prophylactic, he uses the (tincture of?) cannabis indica, a few drops thrice daily for some weeks. Of guarana he says:

"Thirdly, guarana has been introduced to our notice as a remedy for sick headache, and here, again, we have a very valuable addition to our Pharmacopœia. In many instances, especially those of ladies, I have had the most positive assurance given to me of the power of this drug in arresting headache, so that not the slightest doubt can be entertained of its immense value. A dose is usually taken when the headache is approaching; and if this is not quickly successful in arresting it, a second powder is swallowed; after an hour or so, if the remedy is to be useful, the headache has disappeared. I know of several cases in which the greatest enthusiasm is expressed by patients as to its merits. At the same time, I am constantly hearing of cases where it has failed. I am now trying it in smaller doses by daily administration.

"I feel certain that these three drugs—bromide of potassium, cannabis indica, and guarana—constitute a most important addition to our nervine medicines, and that in them we have remedies against a terrible complaint which, a few years ago, constituted the opprobrium of medicine. I might say that I know of cases where galvanism has very speedily cured a pain in the head; and I can call to mind the case of a lady, where the application of the bisulphide of carbon invariably relieved the most severe headaches."

FORMULÆ.—James W. Long (*American Journal of Pharmacy*) gives the following, which he says he is confident have merit in them. One is much used in the lumber camps of Michigan, among the lumbermen, for private diseases:

℞.—Balsam copaiba, fl. ℥ss.; sweet spts. nitre, fl. ℥ss.; gum acacia, ℥ss.; pulv. cubebs, ℥ss.; Venice turpentine, fl. ℥ss.; pulv. podophyllum, ℥ss.; gin, q. s. ft., fl. ℥xii. M. Sig., teaspoonful three times a day.

The following is an excellent wash for cleaning dandruff from the hair and cleaning the scalp:

℞.—Sapon. castil., finely shaved, one teaspoonful; aquæ ammoniæ, f. ʒi.; alcoholis, fl. ℥v.; aquæ coloniensis et spiritus myrciæ in partes æqualles, q. s. ft., fl. ℥viii.

This should be poured on the head, followed by warm water (soft water); the result will be, on washing, a copious lather and a smarting sensation to the person operated on. Rub this well into the hair. Finally rinse with warm water and afterwards with cold water. If the head is very much clogged with dirt, the hair will come out plentifully, but the scalp will become white and perfectly clean. This is for a cleaner of the hair. Should the hair continue to come out on combing (which, however, will not generally happen), use the hair restorative given by Professor Parrish, on p. 312, third edition of Parrish's Pharmacy. As there is more than one on that page, we take the liberty of reproducing it here:

Take of castor oil, f. ℥vi.; alcohol, f. ℥xxvi. Dissolve. Then add tinct. cantharides (made with strong alcohol), f. ʒi.; ess. jessamine (or other perfume), fl. ʒiss. M.

Both of these preparations have been tried, and the result has been a clean scalp, a thorough removal of dead hair, followed by an increased growth of healthy, soft hair.

INGROWING NAIL.—I desire to add a mite to the evidence repeatedly given in the *Journal*, that the removal of the nail (to my knowledge not always successful) is unnecessary.

About twenty years ago, I applied a bit of compressed sponge to afford temporary relief, and was delighted to find that it effected a radical cure. I make the sponge as solid as leather, by wetting and then winding a string very tightly round it and drying it thoroughly. Of this, I cut a small pyramidal piece, less than a grain of rice. This I insert beneath the nail, and secure it by strips of adhesive plaster, applied longitudinally, to avoid compression. The sponge soon becomes moist and swollen, keeping the nail from the irritated flesh. Any granulations should previously be destroyed with strong nitric acid. I have adopted this plan upon many occasions, and have never found it to fail.—*Benjamin Blower.*

HYDROPHOBIA.—The Council of Hygiene, Bordeaux, France, with the view of preventing the spread of hydrophobia, has published the following instructions with regard to dogs: "A short time, sometimes two days, after the madness has seized the dog, it creates disturbances in the usual condition of the animal which it is indispensable to know. 1. There is agitation and restlessness; the dog turns himself continually in his kennel. If he be at liberty, he goes and comes, and seems to be seeking something; then he remains motionless, as if waiting; he starts, bites the air, seems as if he would catch a fly, and dashes himself, barking and howling, against the wall. The voice of his master dissipates these hallucinations; the dog obeys, but slowly, with hesitation, as if with regret. 2. He does not try to bite; he is gentle, even affectionate, and he eats and drinks; but he gnaws his litter, the ends of the curtains, the padding of cushions, the coverlids of beds, the carpets, etc. 3. By the movement of his paws about the sides of his open mouth, one might think he was wishing to free his throat of a bone. 4. His voice has undergone such a change that it is impossible not to be struck by it. 5. The dog begins to fight with other dogs; this is decidedly a characteristic sign, if the dog be generally of a peaceful nature. The numbers three, four and five indicate an already very advanced period of the disease, and the time is at hand when man will be exposed to the dangerous fits of the animals if immediate measures are not taken. These measures are to chain him up as dangerous, or, better still, destroy him."

These instruction are to be published at intervals in newspapers, and also printed on the back of the notice for the dog tax, on the back of the receipt for this tax, and finally on the back of the permission for hunting.—*Western Medical Advance.*

TYPHO-MALARIAL FEVER.—Dr. D. W. Hand, of St. Paul, Minnesota, (*Northwestern Medical and Surgical Journal*), regards the fever which has been so prevalent in St. Paul, for the past six months, of a typho-malarial character. The fatality was not great, although the epidemic was widespread. In his own practice he states that he saw at least one hundred cases, with only three deaths. Is was found that quinine, as an antiperiodic, was useless; as a tonic and stimulant, however, it was useful; and the treatment found best adapted to most of these cases was quinine, one grain twice daily; dilute hydrochloric acid, ten to twenty drops every four to six hours; with an opiate at night to produce sleep and control the diarrhœa. The necessity of keeping up nutrition by milk, beef-tea, oat-meal or flour gruel, etc., was insisted on, and was an important part of the treatment. Stimulants were rarely required.

Dr. G. M. Humphry, of Cambridge, in a clinical lecture published in the *Lancet*, says: "Whether there is much pain or little, whether the patient sleeps or does not sleep, I refrain from giving opium or any other sedative. Pain, even severe and long-continued, though hard to bear, does not seem to do the body much harm. I have often been surprised how little wear and damage it does—not so much, I think, as the sedative which is given for its relief; and the exhaustion of a sleepless, restless night or period is usually followed by sleep, which is more likely to occur naturally at an early period if sedatives have not been used. Under ordinary circumstances, a restless night is less damaging than a night of sleep induced by a sedative. The patient commonly wakes from the latter paler, weaker, lower, more enervated, and less able to bear pain. I have long had this conviction; indeed, throughout life I have acted upon it. It may be regarded as a prejudice; but I think the patients recover better and quicker, and are less liable to pyæmia, erysipelas, and other unfavorable sequelæ of operations when thus treated. These, at any rate, we seldom see in Aldenbrooke's Hospital; and our immunity from them is, I think, partly due to the practice of non-interference after operations so generally followed in this hospital. You will understand that I make a distinction between the use of opium as a remedy for disease and as a means of relieving pain after operations. In the former case, it is a very valuable medicine under many circumstances—one of the most valuable we possess. But in the latter case, there is no disease; the pain is merely a natural consequence of the physical injury which has been sustained, or of the local changes which are taking place as a consequence of the injury. The condition will generally subside after a short time, and it is much better to allow it to do so, unless there is some special reason for interference.—*Medical Times.*

To Prevent Pitting in Small-Pox.—In a case recently treated, in which the eruption so completely covered the face that it was almost impossible to place the point of the finger on it without touching it in one or more places, Dr. J. C. Whitehill, of St. Louis, Missouri, (*Medical Archives*), succeeded in *absorbing the "pocks" completely*, by anointing the face freely with a solution of carbolic acid ℈j., and soda bisulph. ʒij., in an ounce of pure fresh glycerin, and causing each vesicle, as soon as formed, to be punctured with a finely-pointed hard wood, and some of the solution introduced. At the same time light was excluded, as far as possible, from the room, and a liniment of croton-oil used over the chest as a revulsive. Not a "pit" was formed on the face.—*New York Medical Record.*

Apomorphine as an Emetic (M. Loeb, of Worms: *Berliner Klin. Wochenschr.*, No. 33, 1872).—The investigations of Siebert, Riegel and Bochm concerning the nature of this drug as an emetic, induced Loeb to try its effects in the case of a young man who had accidentally swallowed a large draught of alcoholic solution of oil of bitter almonds. The author says: "I was called to see the patient half an hour after the accident, and decided that an immediate emetic must be given. I therefore injected into the region of the stomach 0.008 of a fresh solution of apomorphine. In eight minutes, after repeated gapings on the part of the patient, copious emesis of a mass smelling strongly of hydrocyanic acid occurred. This recurred in five minutes. Then the patient recovered rapidly."

The success of this treatment is ascribed to the promptness of its action.

As a rule, no more than 0.008 of a solution of apomorphine, made in the strength of 1.12 : 4.0, is to be administered. This usually produces in adults two attacks of emesis. In a child thirteen months old, to whom the author gave 0.002 of a solution of this drug, alarming symptoms followed the first attack of vomiting.

(The above report is literally translated; the precise quantities used are not given, but probably 0.008 of a cubic metre,=8 cubic millimetres, or a few minims, is meant.—*Phil. Medical Times.*

Sulphate of Cinchona.—M. Briquet, at a recent meeting of the Paris Academy of Medicine, gave his conclusions with regard to the value of this drug, as based upon eight hundred and ninety-three authenticated cases which had been treated successfully with it since the time of Magendie and Chomel.

Its success has been especially marked in cases of intermittent fever of medium intensity. Furthermore, it arrests the paroxysms of typhoid, mitigates the symptoms of intermittent neuralgia, and is of great benefit in articular rheumatism. Dr. Briquet lays great stress upon the mode of administering the drug. It should be given in watery solution in doses of from fifty centigrammes to one gramme (eight to fifteen grains), according to the intensity of the fever. The whole dose must not be given at once, but must be divided over five or six hours, and it is extremely important that the substance should be taken during the apyretic interval, and at least eight or ten hours before the return of the fit.

Tracheotomy.—Dr. George Buchanan, Lecturer on Clinical Surgery, Glasgow Royal Infirmary, in a recent clinical lecture on "Tracheotomy," states that he has performed this operation in thirty-nine hopeless cases; of these, thirteen recovered, or one out of every three operated on.

Sulpho-Vinate of Soda.—This salt, first recommended by Rabuteau, is the subject of some remarks by the *Gazette Hebdomadaire*, in which the fear is expressed that certain poisonous ingredients may enter it in its manufacture. It is made by first mixing alcohol and sulphuric acid, with an excess of the latter, thus producing sulpho-vinic acid. This is saturated with carbonate of baryta, producing an insoluble phosphate of baryta, and a soluble sulpho-vinate in solution. To this solution is added sulphate of soda, or carbonate of soda, which gives the sulpho-vinate of soda together with carbonate of baryta. The latter salt is removed by an ulterior process. If the decomposition of the sulpho-vinate of baryta should not be complete, a portion of it will remain in the preparation, and give it a poisonous quality. Besides, arsenic may enter into the composition of the carbonate of baryta. If, however, the sulpho-vinate of soda be carefully prepared, it has two advantages over sulphate of soda and sulphate of magnesia as a purgative; a smaller dose is required, five or six drachms being a sufficient cathartic for an adult; and it is less unpleasant to the taste, especially if it be dissolved in the same quantity of water required for the other salts.—*Pacific Medical Journal.*

Puncture of the Abdomen for the Relief of Tympanitis.—The *Dublin Quarterly Journal of Medical Science* mentions three cases in which marked relief was afforded by this operation. In one the distension was caused by the pressure of an ovarian tumor on the intestine. The puncture was made in the cœcal region, and was repeated daily, more than fifty times, at the request of the patient. At the autopsy no traces of the punctures could be observed. The second case was that of a man sixty-one years old. Eight punctures were made in fourteen days, with great relief and no unpleasant results. In another case, reported in the *Practitioner*, the operation was performed upon a patient with double pneumonia. The punctures, two in number, were made over the transverse and descending colon, and gave great relief. The patient, however, died of pneumonia, but no traces of the puncture could be found after death, except on the surface of the body. The instrument used was an exploring tochar (*m.* 1 *Weiss*).

Nitrate of Silver in Epilepsy and Chorea.—In 1802, Dr. Sims, of London, read a paper before the Medical Society of London, on the great value of this remedy in epilepsy and chorea. This paper, it is believed, contains the earliest notice of the efficacy of this salt in these diseases.

The value of elaterium as a purgative in dropsy was mentioned by Dr. Sims as early as 1798.—*New York Medical Record.*

Balsam of Copaiba in Small-Pox and Scarlatina (*Medical Times and Gazette*, February 17).—From our knowledge of the effects of the balsam on the skin and mucous membranes, Dr. A. Rowland, Visiting Physician and Surgeon to the Marine and Emigrant Hospital, etc., Quebec, was induced to try it, in four or five drop doses, mixed in ʒ ij. sirup and ʒ ij. mucilage of gum Arabic, three or four times a day, in the confluent small-pox of a person who had never been vaccinated. It caused no nausea, but, on the contrary, created a keen appetite, which continued till recovery. No pitting took place, and no local application was used but glycerin and water. Dr. Rowland tried the same mixture in scarlet fever, with most satisfactory results. Under its use, the tongue and sore-throat got rapidly clean and well, followed by a keen appetite, and by none of the usual sequelæ. The secretion of urine was copious, and began to increase in quantity after two or three doses. At first it was the color of ale and a little ropy, but by the third day quite clear and normal. The the author's theory of the action of the remedy is, that it alters or destroys the character of the virus, and eliminates it out of the system by the skin and kidneys more particularly; for the recoveries have been unusually rapid. In both cases Dr. Rowland prescribed milk, beef-tea, wine, and spirits, according to need.

Posture as an Important Adjunct in the Treatment of Diarrœa and Dysentery.—E. P. Sale, M.D., of Aberdeen, Mississippi, writes: Rest, as is well known, is the *sine qua non* of inflammations, and in order to secure it in the above intestinal diseases, I have for some time past directed patients to assume a *prone position* with a compress—*e. g.*, a pillow or bolster—placed under the abdomen, the amount of compression to be governed by the degree of tenderness. I conceive this method to approach in effect the splints, used to retain fixture of limbs. The compress to a great extent prevents peristaltic action of the intestines, especially when used in connection with opiates. The position renders respiration more thoracic, thereby preventing movement of the intestines, which is consequent of abdominal respiration, and tormina in a great degree is relieved.

Treatment of Chilblains.—Mr. Fergus recommends sulphurous acid in this affection. It should be applied with a camel's hair brush, or by means of a spray producer. One application of this usually effects a cure. The acid should be used pure. A good wash for hands or feet affected with chilblains is sulphurous acid, three parts; glycerin, one part; and water, one part. The acid will be found particularly useful in the irritating, tormenting stage of chilblains.—*Cincinnati Medical Repertory.*

A GOOD LESSON ON DIAGNOSIS.—In a clinical lecture in the Liverpool Royal Infirmary, reported in the *London Lancet*, Dr. Waters makes the following very sensible remarks:

"It may seem to you an easy matter to make out the nature of a case. A man of experience will put a few leading questions, make perhaps only a slight physical examination, and at once pronounce a correct opinion of the existing ailment; and you may imagine that you will readily be able to do the same. But do not be mistaken. This power of rapid diagnosis has been the result of long observation and great painstaking; and you will find even that the man of the most matured judgment will often spend a long time over a case—will examine carefully all its details—before he will venture an opinion of its nature. If you wish to attain to the power of rapid diagnosis—of rapid diagnosis in ordinary cases—you must begin by examining with the greatest care every detail of a series of cases which are the best-marked instances of their kind. The study of these will prepare you to understand the varieties and complications which so constantly present themselves at the bedside."—*Pacific Medical Journal.*

PILLS OF FERROUS OXIDE.—W. Kirchman has, for the last ten years, prepared the following pills, which have been used with favorable results by several physicians: ℞. Ferri sulphatis, 120 grains; magnesiæ ustæ, 20 grains; glycerini, 15 drops. M. ft. pilul. No. 60. The pills are well adapted for sugar coating; placed in water, they dissolve at once, leaving ferrous oxide behind. Ferrous and magnssium sulphate containing the same amount of water of crystallization, the above formula yields a good mass without any further addition. The glycerin prevents the magnesia salt from drying, the fine crystalline mass of which envelopes the ferrous oxide so completely as to prevent all oxidation, even when kept for years.—*Archiv d. Ph.*, 1872, *September*, 231.—*American Journal of Pharmacy.*

THE LOCAL AND SUPERFICIAL ANÆSTHETIC EFFECTS OF THE PHENOLS.—According to Dr. Edward R. Squibb, of Brooklyn, New York, (*New York Medical Journal*), the best of these is cresol or cresylic acid, and next phenol or the crystallized carbolic acid. But practically the cheap mixture of the two, called coal-tar creosote or impure carbolic acid, is as good as either. The prompt and complete effect of very dilute aqueous solutions of this creosote upon the pain of burns, erysipelas, etc., led him to infer peculiar anæsthetic properties many years ago, and the numbneess or insensibility produced upon the hands by handling it confirmed the idea.

Preparations of Eucalyptus Globulus.—*L'Union Pharmaceutique*, 1872, September, publishes the following formulas:

*Sirup of Eucalyptus.*—Distilled water of eucalyptus, 500 parts; sugar, 950 parts. Dissolve without heat and filter.

*Tincture of Eucalyptus.*—Eucalyptus leaves, dried and cut, one part; alcohol of 80 per cent., five parts. Digest for six days and filter.

*Wine of Eucalyptus.*—Eucalyptus leaves, dried and cut, 30 parts; alcohol of 60 per cent., 60 parts; white wine, 1,000 parts. Macerate the leaves in the alcohol for 24 hours, add the wine, macerate for ten days, express and filter.

*Extract of Eucalyptus.*—Eucalyptus leaves, dried and cut, 1,000 parts; water, 3,000 parts. Obtain by distillation the volatile oil. Of the residue in the still, prepare an aqueous extract, which treat with 1,000 parts of alcohol of 60 per cent., filter, concentrate the alcoholic liquid to the consistence of an extract, to which, while cooling, add the volatile oil and mix intimately.—*American Journal of Pharmacy.*

Sulphate of Iron in Suppuration.—A child burned nearly over the whole body was recently brought to the Child's Hospital at Lausanne. The suppuration was so abundant that the ward in which the patient lay became uninhabitable. The child was then placed in a bath containing two handsful of sulphate of iron. The cessation of pain was almost immediate. After repeating the bath twice a day, for fifteen or twenty minutes at a time, the suppuration moderated, the fetid odor disappeared, and the patient rapidly recovered.—*Boston Journal of Chemistry.*

Cancer of the Liver.—Dr. N. S. Davis, of Chicago, Illinois, (*Chicago Medical Examiner*), has realized, in the treatment of this disease, the most benefit from some of the arsenical preparations with conium. The following is a good formula: ℞. Arsenite of soda, gr. iv.; ext. conium, ʒ iss. Make a mass and divide into sixty pills, one three or four times daily. Among the best anodynes to relieve pain are fluid ext. hops and lettuce. In this way the fatal result may be delayed.

Tincture of Calendula in Phagedæna.—Thomas Kennard, of St. Louis, Missouri, (*Medical Archives*), advocates the use of the tincture of calendula (garden marigold) as a local application in phagedæna. He has used it in preference to aromatic wine as a dressing for syphilitic sores for many years. It is also recommended as a stimulant and healing application for lacerated wounds, ulcers, or any breach of continuity of the surface.

TREATMENT OF THE INFLAMED BREASTS OF NURSES.—The method here recommended is so simple that no one need hesitate to adopt it, provided he is called in before the mischief has reached a certain degree of development. It is well-known that engorgements of the mammary glands are frequently caused by chapped nipple. The inflammation of the skin extends directly into the ducts, exudations take place by which some of the ducts are plugged up, the milk is pent in, and hence the engorgement. If, now, in such a case, the breast be surrounded with the hands, and pressure made in the direction of the nipple, a thin, transparent, whitish vesicle is caused, by the milk accumulating behind the closed orifices of the ducts. It is necessary, then, to do this, and, having done it, the next thing is to prick the vesicle with a needle, to remove any epithelial scales which may be present, and to apply the infant. If time has not been lost unnecessarily, the relief is almost immediate, and pain and tumefaction disappear in a few minutes; but even when it is otherwise, the relief is very marked, and by repeating the process a few times, the sufferer is relieved altogether. *M. R.*

PURIFICATION OF CHLORAL HYDRATE.—Prof. E. A. Flueckiger states (*Phar. Cent. Hall*) that much of the commercial chloral hydrate is impure, and unfit for use until purified. It is usually met with in irregular masses containing moisture, and having a sharp, pungent odor, indicating partial decomposition. The salt may be readily purified by dissolving it in *pure* bisulphide of carbon, (soluble in forty-five parts of the menstruum at 18° C), and allowing the salt to crystallize by the spontaneous evaporation of the solvent. The product appears in colorless prismatic crystals, not hygroscopic, and free from acid reaction.

USE OF CAMPHOR IN HOSPITAL GANGRENE.—M. Netter, of the Military Hospital of Rennes, has found camphor, in powder, very efficacious in *hospital gangrene,* by sprinkling it abundantly over the wound. In his service, as well as in that of M. Aubry, surgeon of the same institution, they had treated this affection by the usual means—chloride of iron and carbolic acid with alcohol—but without success. By using very freely the powdered camphor, three patients were successfully treated, and in forty-eight hours the disease disappeared from the hospital.—*N. Y. Med. Journal.*

METHOD OF MAKING LEECHES BITE.—Place them in a mixture of two parts of wine and one of water; they are in a few minutes very active, and take hold instantly; and if they are gorged with blood, they disgorge themselves and will draw again.

Tincture of Iodine in Cholera Infantum and Dysentery.—Dr. Gilliam (*Clinic*) says: "As iodine has the property of arresting transudations, and as it is of particular value in many inflammatory affections of the skin, and as the skin and mucous membrane are very analogous in structure, *a priori*, it would seem probable that iodine might be of service in the above-named affections. I have tried it in a case of cholera infantum and in a case of dysentery. It seemed to yield good results in the former, and eventuated in a cure. In the latter, its influence was not so marked, though seemingly beneficial. I am now trying it in a case of dysentery, and am encouraged both by its effect on the patient and a statement going the rounds of the journals to the effect that a very obstinate case of vomiting has been checked by the administration of ten-drop doses on sugar, where everything else had failed. I give it in doses of from eight to fifteen drops every four hours to an adult, either on sugar on in glycerin."

Treatment of Enlarged Tonsils.—Dr. Rumbold, St. Louis, Missouri, (*Medical Archives*), has treated successfully a number of cases of enlarged tonsils by injecting the glands, by means of a hypodermic syringe, with a solution of iodine—iodine, gr. ij.; pot. iod., ℈ ij.; aquæ, ℥ j. Generally, a slight inflammation followed the injection, but soon subsided. From twelve to seventeen injections—ordinarity two a week—were sufficient to reduce the gland to its normal condition. The advantage claimed for this mode of treatment was saving the substance and function of the gland.

The Therapeutic Value of Gelseminum.—Dr. Philip C. Williams, Baltimore, Maryland, (*Baltimore Medical Journal*), in an extended paper on the value of "Gelseminum," believes that it will cure all pure, simple neuralgias of the cerebral system with promptness and efficiency; that it will relieve cerebral congestion; that it controls maniacal excitement, and a variety of conditions resulting from derangements of the central nervous system. For these and analogous disorders he recommends gelseminum as a most powerful and efficient remedy.

Treatment of Chronic Gastralia.—M. Valleix has derived the greatest benefit from the employment of small doses of acetate of morphia. He prescribes it immediately after a meal, and in this way he has relieved cases which had resisted all other treatment. He orders gr. i. of the acetate in ℨ xxx. of distilled water and ℨ ix. of sirup, and directs a teaspoonful to be taken immediately after each meal. Under the use of these small doses, the bowels, so far from becoming constipated, are better regulated.

Lactic Acid in Dyspepsia.—Dr. C. Handfield Jones advises the use of lactic acid in dyspepsia. He has chiefly given it in cases of irritative dyspepsia, where the digestion was painful and imperfect, and had been so for some time; he does not advise its use at the commencement of the treatment in a severe case, but only after irritation and vascular erethism is somewhat reduced. It should be employed in doses of fifteen or twenty minims in a half-ounce of water, and taken *at* meal-times; he states that it seems to mingle with the food, and to supply one of the constituents of healthy gastric juice, which is probably imperfectly produced. Its use need not be confined to cases of dyspepsia, but may be extended to all cases where it is desirable to improve the tone and power of the stomach. It is pleasant, occupies but little space, and the only objection to its use is its present high price; but if much employed, it could probably be obtained cheaply.

Treatment of Psoriasis.—We observe in a recent number of the *British Medical Journal* that that leading dermatologist, Dr. Tilbury Fox, thinks that the treatment of psoriasis by arsenic internally, and tarry preparations externally, is erroneous, and much too generally employed. For his own part, he has almost entirely given up this plan of treatment, except in certain chronic cases and where there is a syphilitic taint, when he found Donovan's solution of great value. In all other cases, he relies mainly upon soothing applications locally, viz.: wet packing, alkaline and sitz baths, and oily preparations; and internally, remedies in accordance with the constitutional diathesis. This plan of treatment is especially successful in acute cases occurring in young children. Dr. Fox lays great stress upon psoriasis being treated on the same principles as other cases, with due regard to constitutional and other causes likely to affect and modify it.—*Medical and Surgical Reporter.*

A mixture of pulv. steatite (soapstone) two parts, and hyd. chlor. mitis one part, is the most elegant and effective dry application to the chafed skin of infants. Dry hyd. chlor. mitis, applied once or twice a day to tumid and hemorrhoids situated about the anus, rarely fails to cure them in a few days.—*Dr. A. S. Hudson, in the Pacific Medical and Surgical Journal.*

Nux Vomica in the Diarrhœa of Exhaustion.—℞. Alcoholic extract of nux vomica, (not officinal, but prepared by most wholesale druggists), gr. ss.; rhubarb, gr. ss.; saccharated carbonate of iron, gr. j.; blue pill, gr. ss.; opium, gr. ⅛—made into a pill, and taken three times daily. In many cases the opium may be omitted.

Treatment of Gonorrhœa by Tanno-Glycerin Paste.—Dr. Tomowitz, K. K. Regiments arzt, Austrian army, reports the successful employment of Schuster's (Aix la-Chappelle) tanno-glycerin paste in somewhat different form for syphilis and gonorrhœa. His formula is as follows: ℞. Tannini puri, ℥ ss.; opii perverisati, gr. iv.; glycerinæ, q. s. ut ft. pasta. Some fifty to sixty drops of glycerin are requisite to bring the paste to a proper consistency. A sound or elastic bougie is dipped into the paste, warmed over a stove or spirit lamp, and thus smeared, is introduced into the orificium penis to the fossa navicularis, where it is held for five minutes. This operation is repeated three times a day. In gleet, the catheter or bougie is carried back to the bladder and slowly withdrawn, so as to bring the paste into contact with every surface of the urethra. Even in acute cases, the pain is but very slight.—*Allg. Militarztl. Zeit.—Clinic.*

Phtheiriasis.—W. T. Carter, M.D., Louisville, Kentucky, (*American Practitioner*, October, 1872), recommends the following solution for the destruction of parasites: Corrosive sublimate, gr. xij.; alcohol, ℥ iv.; oil of bergamot, gtts. vj. Mix and add water, ℥ iijss. This mixture should be thoroughly applied to every part of the body infested. The first application will, in the majority of cases, cause the death of every accessible louse; but it should be continued twice daily for at least one week, in order that none may escape. In that particular condition of the system described so well by Dr. McCall Anderson, in which lice multiply on the body in such numbers and with such astonishing rapidity, the iodide of potassium alone, or in combination with prussic acid, given internally, has yielded the most satisfactory results.

Carbolic Acid in Obstinate Leucorrhœa.—Dr. Whitaker, of Cincinnati, (*Cincinnati Lancet and Observer*), speaks highly of carbolic acid in the treatment of obstinate leucorrhœa. The application is made upon the cotton-wrapped sound. The instrument, so enveloped, is first introduced and the uterine surface cleansed; a new layer of cotton is substituted, medicated by immersion in a solution of proper strength, and applied to every portion of the uterine cavity.

Citrate of Iron and Suephate of Quinine in Chlorosis and Other Anæmic States.—Take of citrate of iron, ʒii.; sulphate of quinia, ʒss; water, f℥i. Mix, and direct from twenty to thirty drops for the dose, in syrup and water, after each meal, within half an hour of the breakfast, dinner or supper, so that it may be carried with the chyme along the course of the bowels.

Foreign Body in Interior of Eye.—Professor S. D. Gross (*Michigan University Medical Journal*) recommends the following "treatment when a foreign substance, lodged in the interior of the eye, cannot be extracted. Where useful vision yet remains, and the foreign body is creating no irritation, the case should be kept under close observation, and the patient cautioned to seek aid upon the appearance of symptoms of irritation in the sound or uninjured eye. Where useful vision is destroyed, and the foreign body yet remains, giving rise to destructive inflammation, the eyeball should be extirpated to remove the cause of a probable sympathetic ophthalmia in the sound eye. Although dissection has shown that foreign bodies in the eye, as shot, pieces of iron and gun caps, may become encysted, yet such an event is extremely rare, and does not afford immunity against future attacks of inflammation. As long, in fact, as the extraneous substance remains, it is liable at any time, whether free or adherent, to provoke suffering and disease, and to induce sympathetic inflammation in the sound eye.—*Med. Cosmos.*

Dugong Oil.—Attention (*British Medical Journal*) has been recalled, by the contents of the Queensland Annexe at the International Exhibition, to the medical uses of dugong oil. It has been declared by more than one medical practitioner, following Dr. Holt, of Brisbane, to possess all the nutritive properties of cod-liver oil, and to be equally useful in all the forms of tuberculous and wasting diseases which are benefited by the administration of cod-liver oil; it is alleged to possess an actually agreeable flavor, to be pleasant as an article of food, and to be acceptable to those whose stomachs reject cod-liver oil. At a recent dinner in the Annexe, the pastry was made with dugong oil, and pronounced to be excellent.

Nocturnal Incontinence Treated by Chloral.—Dr. Girolamo Leonardi (*L'Ippocratico*, 10th July) has found chloral useful even in adults long affected with nocturnal incontinence of urine, in the dose of one gramme dissolved in sixty grammes of water at bed-time. The patient is not to drink much at supper. He mentions one case of rapid cure in a young scrofulous male, aged twenty-four, and another in a boy aged seven. The cure was without delay.

External Use of Fuming Nitric Acid.—Professor Patruban (*Memorabilien*, June, 1872) says that this heroic caustic is indicated on account of its rapidity and drying-up of the tissues. It stops all effusion of blood, and leaves good scars. He uses it for warts and other protuberances of the skin, as condylomata, hemorrhoids, and tumors of the nature of nævus.—*Doctor.*

Typhoid Fever.—Arnica may be valuable in collapse, but where there is lung complication, with increased action of the heart, and rapidity of breathing, the tincture of arnica, in doses of fifteen to twenty drops every three or four hours, will be found exceedingly useful, reducing the pulse with almost the certainty of veratrum, without its depressing effect. Here, too, ipecac is often of great value. Arsenic, too, (in the form of Fowler's solution), where the tongue is dark and dry, with red edges, the secretions suppressed, the stomach irritable, the bowels flatulent, will be found in many cases exceedingly useful. It is well borne in doses of three or four drops every six hours.—*Clinic.*

Arsenic in Disease of the Liver.—In a case of disease of the liver, (recorded by Dr. de Moura in the *Gaceta Medica de Bahia*), in a patient of cachectic habit, with pallor of the surface, polyuria, want of appetite, and tumefaction of the liver, and in which analoid degeneration of the organ through syphilitic causes was diagnosed, the administration of arsenic was completely successful, after iron, the vegetable tonics, iodide of potassium, and corrosive sublimate had all failed.

Tincture of Chloride of Iron for Corns.—Dr. C. Barber states (*Lyon Médicale*) that he has cured three cases of corns on the toes by the application of a drop of the tincture of chloride of iron applied on corns night and morning. This application was continued for fifteen days in one case, when the corns from which the patient had suffered for thirty or forty years were entirely destroyed, and pressure on the part gave not the least uneasiness.

Extracts in Powder (*Boston Medical and Surgical Journal.*) It is sometimes very desirable to administer the solid extracts (say extract of hyoscyamus) in the form of a powder. All such extracts may be reduced to fine powder by patiently rubbing them (say two or more hours) in a mortar with an equal quantity, by weight, of calcined magnesia. Extract of gentian yields the slowest to this manipulation.

Erysipelas.—Dr. Reamy (*Clinic*) says that in cases where large doses of mur. tr. iron would not be borne by the stomach, he withholds the iron for a time, giving a few doses of potass. bromid., which he finds almost invariably to give tolerance to the stomach, when the iron can be resumed in full doses.

Vermifuge Sirup.—Extract spigelia maryl., fl. ℥ viii.; sirup sennæ compos., (Lond. Ph.), ℔. ii. Mix while hot, and evaporate to a proper consistence. Dose, a small teaspoonful for a child one year old.

A New Mode of Administering Copaiba.—Dr. Wehner, of Pennsylvania, has obtained, in chronic cases of gonorrhœa, excellent results from copaiba without any of its nauseating inconveniences, by administering the drug combined with opium in the form of rectal suppositories. He recommends the following formula: Copaiba, six ounces; opium powder, five grains; oil of theobroma and spermaceti, of each an ounce and a half; from forty to sixty grains of white wax, for twelve suppositories; one to be introduced morning and night.

The Endermic Application of Belladonna for Neuralgia.—Prof. Lippich, of Padua, employs the following formula when he desires to have recourse to the ext. of belladonna: Take mucilage of gum Arabic, 300 grains; extract of belladonna, eight grains. Mix well together. Apply upon the surface previously denuded by a blister. M. Lippich in this manner has seen rapid and complete success in various cases of rheumatismal lumbago, cephelagia, etc.

Strychnia in Albuminuria.—Brignolia, in *Lo Sperimentale*, besides recommending nux vomica in various neuroses, gastralgia, dyspepsia, carbiac palpitations, periodic cough, etc., states that he has observed it to have a marked effect in retarding the progress of albuminuria, especially the scarlatinal form with anasarca. He cites twelve cases of complete recovery.

The *Medical Times and Gazette* (September 3d and October 1st, 1870) contains articles commendatory of the use of iodine (two grains of iodine and a little iodide of potassium to an ounce of water) as a topical application to wounds, of its use in syphilis as a gargle, and in form of an ointment externally to relieve the osteocopic pains in the shin-bones.—*N. Y. Medical Journal.*

Urticaria from Privy Seats.—In the *Berliner Klinische Wochenschrift,* Dr. Höfling reports he has often observed urticaria around the anus from the use of unclean privies. He regards it as produced by the mould which clings around such places, and which acts as an irritant and local poison.

Nitrite of Amyl in Spasmodic Asthma.—Dr. James A. Duncan, of Toledo, Ohio, relates (*Michigan University Medical Journal*) three cases of spasmodic asthma promptly relieved by a few drops of the nitrite of amyl.

Pill for Gastralgia.—℞. Sub. nitrate of bismuth, ʒii.; ext. belladonna, gr. x. To be made into forty pills, and give one night and morning.

